# The ubiquitin hybrid gene *UBA52* regulates ubiquitination of ribosome and sustains embryonic development

**DOI:** 10.1038/srep36780

**Published:** 2016-11-10

**Authors:** Masanori Kobayashi, Shigeru Oshima, Chiaki Maeyashiki, Yoichi Nibe, Kana Otsubo, Yu Matsuzawa, Yasuhiro Nemoto, Takashi Nagaishi, Ryuichi Okamoto, Kiichiro Tsuchiya, Tetsuya Nakamura, Mamoru Watanabe

**Affiliations:** 1 Department of Gastroenterology and Hepatology, Graduate School, Tokyo Medical and Dental University (TMDU), Tokyo 113-8510, Japan; 2 Center for Stem Cell and Regenerative Medicine, Tokyo Medical and Dental University (TMDU), Tokyo 113-8510, Japan; 3 Department of Advanced Therapeutics for GI Diseases, Tokyo Medical and Dental University (TMDU), Tokyo 113-8510, Japan

## Abstract

Ubiquitination is a crucial post-translational modification; however, the functions of ubiquitin-coding genes remain unclear. *UBA52* encodes a fusion protein comprising ubiquitin at the N-terminus and ribosomal protein L40 (RPL40) at the C-terminus. Here we showed that *Uba52*-deficient mice die during embryogenesis. *UBA52*-deficient cells exhibited normal levels of total ubiquitin. However, *UBA52*-deficient cells displayed decreased protein synthesis and cell-cycle arrest. The overexpression of UBA52 ameliorated the cell-cycle arrest caused by UBA52 deficiency. Surprisingly, RPL40 expression itself is insufficient to regulate cyclin D expression. The cleavage of RPL40 from UBA52 was required for maintaining protein synthesis. Furthermore, we found that RPL40 formed a ribosomal complex with ubiquitin cleaved from UBA52. UBA52 supplies RPL40 and ubiquitin simultaneously to the ribosome. Our study demonstrated that the ubiquitin-coding gene *UBA52* is not just an ubiquitin supplier to the ubiquitin pool but is also a regulator of the ribosomal protein complex. These findings provide novel insights into the regulation of ubiquitin-dependent translation and embryonic development.

Ubiquitination is a common post-translational modification. The 8-kDa protein ubiquitin is covalently attached to one or more lysine (Lys) residues of a substrate protein^1^. Ubiquitin chains can attach at seven ubiquitin Lys residues (K6, K11, K27, K29, K33, K48, and K63) or at the ubiquitin amino-terminal methionine 1 residue (generating linear chains)^2^. Different ubiquitin linkage types have different functions in the cell cycle^3^, nuclear factor kappa-light-chain-enhancer of activated B cells (NF-κB), and cell-death signaling^4^,^5^,^6^. Ubiquitin proteins are encoded by four genes (*UBA52*, *UBA80*, *UBB*, and *UBC*)^7^,^8^. UBB and UBC are polymers of ubiquitin linked in a “head-to-tail” manner. UBA52 and UBA80 comprise a single ubiquitin fused at the C-terminus to ribosomal protein (RP) L40 and S27a, respectively^9^,^10^ (Fig. 1A). UBA52 and UBA80 are mainly post-translationally processed^11^. A pool of free ubiquitin is maintained through synthesis by ubiquitin-coding genes, release from protein-conjugated ubiquitin chains, and release from unanchored ubiquitin chains^12^,^13^. It is assumed that the ubiquitin pool is under strict regulation in order to ensure appropriate responses to different cellular conditions. However, the function of ubiquitin-coding genes as a source of ubiquitin remains unknown.

Genetic approaches have been used to analyze the physiological function of ubiquitin-coding genes. Mice lacking one or both copies of UBB develop normally and are viable at birth^14^. Loss of UBB leads to a progressive degenerative disorder that affects the neurons^15^. In contrast, loss of UBC leads to embryonic death between embryonic days (E)12.5 and E14.5 *in utero*^16^. UBA52 and UBA80 are preferentially overexpressed during hepatoma cell apoptosis^17^. In addition, previous findings suggested that UBA52 functions in the pathogenesis of diabetic nephropathy^18^. Northern hybridization also suggested that UBA52 is expressed in human colorectal carcinoma^19^. However, the physiological functions of the ubiquitin hybrid gene *UBA52* remain unclear.

Ribosome biogenesis and protein synthesis are tightly regulated process linked to other fundamental cellular processes^20^,^21^. Targeted disruption of the ribosomal protein genes (e.g., *Rps19* and *Rps6*) is lethal^22^,^23^. Dysfunctional ribosome biogenesis is associated with developmental defects and an increased risk of cancer^24^,^25^. Recent studies have shown that regulatory 40 S ribosomal ubiquitination is an important phase of translational control^26^. In addition, translation reactions terminate with ub-dependent removal of defective nascent chain^27^,^28^. Nevertheless, the association between ubiquitin-dependent regulation of translation and the ubiquitin-ribosomal hybrid gene *UBA52* remains unclear. To determine the physiological functions of UBA52, we generated mice lacking *UBA52*. The obtained findings highlight the central roles of ubiquitin-coding genes in the regulation of physiological ubiquitin and ribosomal function *in vivo*.

## Results and Discussion

### UBA52 is required for embryonic development

**Figure 1:**
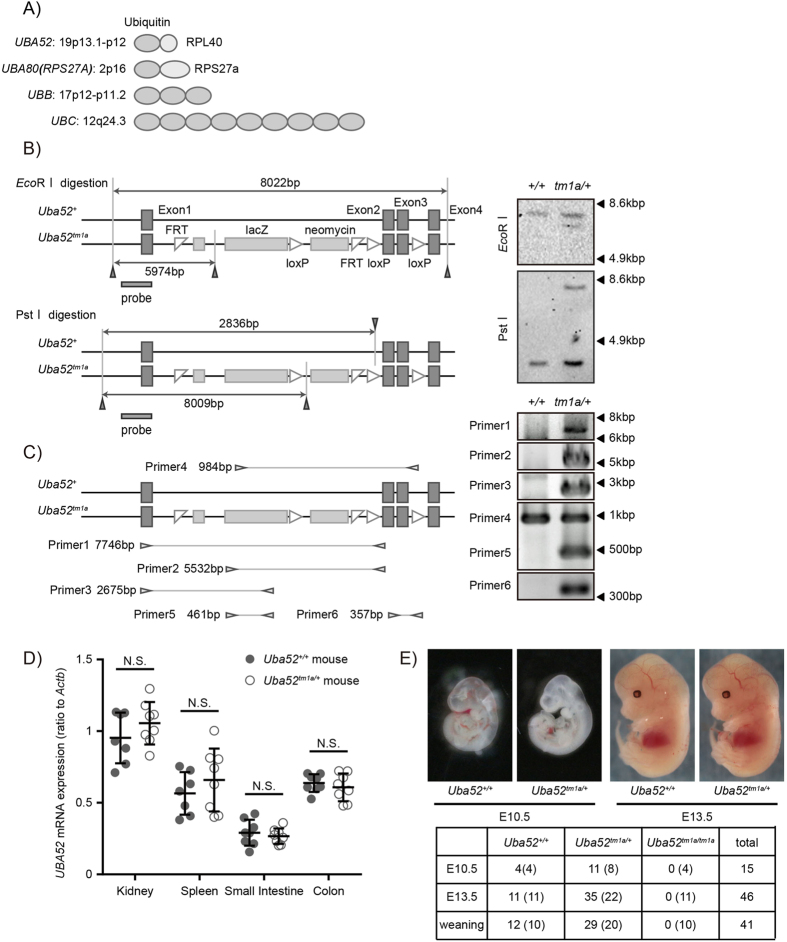
UBA52 is required for embryonic development. (**A**) Diagram of human ubiquitin genes and their chromosomal locations. (**B**) Schematic representation of the gene-targeting construct and screening strategy. Southern blots of *Eco*RI- and *Pst*I-digested genomic DNA from embryonic stem cells showing the targeted allele. The probe was specific for exon 1. (**C**) Polymerase chain reaction (PCR) analysis of genomic DNA obtained from the tails of wild-type (*Uba52*^+/+^) and mutant (*Uba52*^*tm1a*/+^) mice using the PCR primers indicated. (**D**) Quantitative real-time reverse-transcription PCR of *Uba52* mRNA expression in tissues (kidney, spleen, small intestine, and colon) of 3-week-old mice. mRNA levels were normalized to *Actb* levels. *Uba52*^+/+^ (*n* = 8), *Uba52*^*tm1a*/+^ (*n* = 8). Error bars indicate standard deviations. N.S. indicates not significant by two-tailed Student’s *t*-test. (**E**) Numbers of surviving offspring from *Uba52*^*tm1a*/+^ parents. Pictures show embryonic day (**E**) 10.5 and E13.5 littermates. Parentheses: expected numbers based on a Mendelian ratio.

To investigate the potential physiological roles of UBA52, we generated *Uba52*-deficient mice. We confirmed insertion of the *tm1a* cassette into *Uba52* genomic fragment in embryonic stem cells by Southern blotting (Fig. 1B) and in DNA obtained from the tail by polymerase chain reaction (PCR; Fig. 1C). We found that the deletion of one *Uba52* allele in mice did not affect the expression of *Uba52* mRNA (Fig. 1D). To further confirm the *UBA52 tm1a* allele, we consider that aberrant UBA52 proteins may act as dominant-negative molecules. We analyzed the UBA52 protein expression by immunoblotting; the truncated protein was not detected in *Uba52*^*tm1a*/+^ liver lysates (data not shown). However, no live-born *Uba52*-deficient (*uba52*^*tm1a*/*tm1a*^) mice were obtained from intercrossed *Uba52*^*tm1a*/+^ parents (Fig. 1E). Because *Ubc*-deficient mice died at around E13.5, we analyzed *Uba52*-deficient mice at this time. *Uba52*^tm1a/+^ normally developed, but we could not obtain *uba52*^tm1a/tm1a^ at E13.5 (Fig. 1E). *Rps6*-heterozygous embryos die during gastrulation^23^. Therefore, we analyzed at E10.5, but we could not obtain *uba52*^tm1a/tm1a^ at E10.5. Thus, these data indicate that one allele of the *Uba52* gene is enough for development but UBA52 is required for embryonic development.

### UBA52 regulates protein synthesis

**Figure 2:**
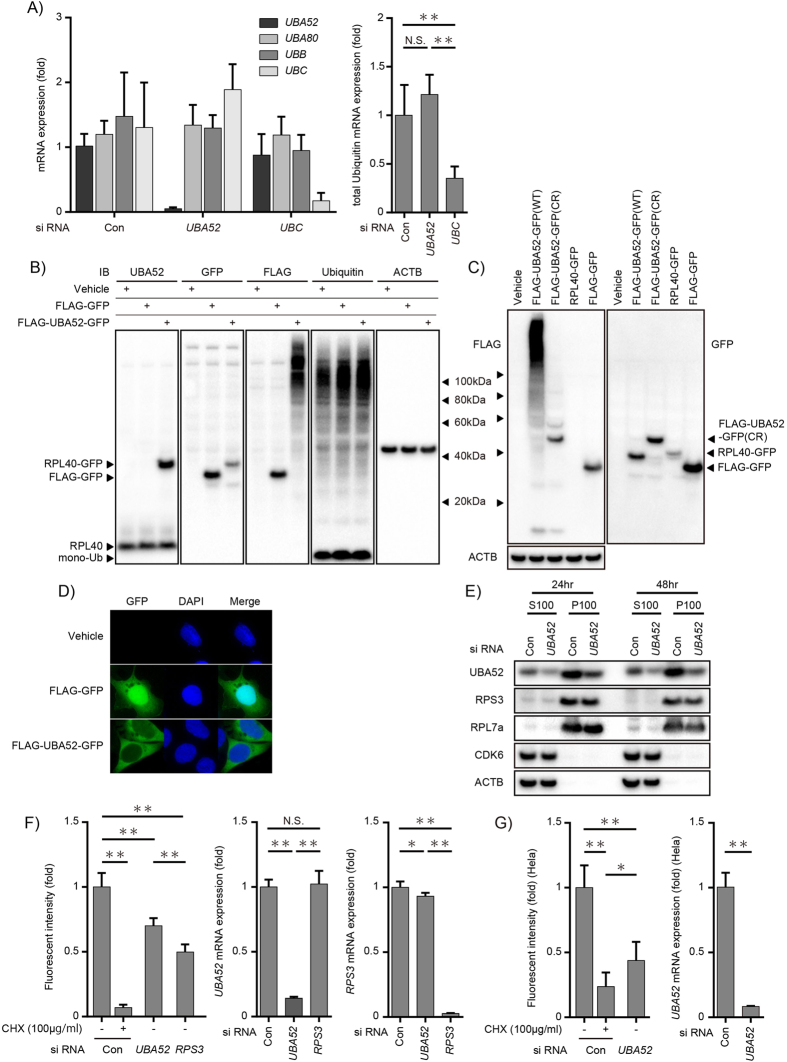
UBA52 regulates protein synthesis. (**A**) Ubiquitin expression in *UBA52*-deficient cells. Total RNA was isolated from DLD-1 cells 24 h after short interfering RNA (siRNA) transfection. *UBC*, *UBB*, *UBA52*, and *UBA80* mRNA levels were measured by quantitative real-time reverse-transcription PCR and normalized to *ACTB* levels. Error bars indicate standard deviations. Data represent three independent experiments (*n* = 8). **P < 0.01 (one-way ANOVA followed by Tukey’s test). (**B**) UBA52 cleavage in DLD-1 cells. FLAG-UBA52-green fluorescent protein (GFP) expression in DLD-1 cells. DLD-1 cells were transfected with FLAG-GFP or FLAG-UBA52-GFP. After 45 h, cells were lysed and immunoblotted for the indicated proteins. Data are representative of more than three independent experiments. (**C**) Cleave-resistant mutants in HEK293T cells. HEK293T cells were transfected with three types of mutation vectors. After 24 h, cells were lysed and immunoblotted for the indicated proteins. G75/76A mutations lead to cleavage resistance (FLAG-UBA52-GFP (CR))^17^. Data are representative of more than three independent experiments. (**D**) UBA52 localization in DLD-1 cells. DLD-1 cells were transfected with FLAG-GFP or FLAG-UBA52-GFP. After 24 h, images were acquired using a confocal laser microscope. Data are representative of three independent experiments. GFP (green) and 4′,6-diamidino-2-phenylindole (blue). (**E**) Subcellular fractions of DLD-1 cells. DLD-1 cells were transfected with a *UBA52* siRNA and lysed for ultracentrifugation at the indicated time. S100 (cytosol) and P100 (crude ribosome pellet) fractions were immunoblotted for the indicated proteins. Data are representative of more than three independent experiments. (**F**,**G**) Protein synthesis in *UBA52*-deficient cells. DLD-1 cells (**F**) or Hela cells (**G**) were transfected with *UBA52* or ribosomal protein (RP) *S3* siRNAs. The cells were treated with cycloheximide (100 μg/ml) for 3 h. Cells were incubated with O-propargyl-puromycin (20 μM) for 30 min and then harvested for the protein synthesis assay. Data are representative of more than two independent experiments. *P < 0.05, **P < 0.01 (one-way ANOVA followed by Tukey’s test). We confirmed knockdown efficiency by quantitative real-time (RT) PCR and normalized to *ACTB* levels. Data are representative of two independent experiments.

To better understand how UBA52 sustains embryonic development, we noted that UBC is essential for fetal development^16^. Given that *UBA52* is a ubiquitin hybrid gene, we hypothesized that UBA52 regulates the total ubiquitin mRNA expression. To investigate this possibility, we used a short interfering RNA (siRNA) approach for reducing UBA52 expression in a colon cancer cell line (DLD-1). Acute knockdown of *UBA52* did not affect the total ubiquitin mRNA levels. Conversely, knockdown of *UBC* reduced the total amount of ubiquitin (Fig. 2A). Our finding that *UBA52*-deficient cells display similar levels of total ubiquitin mRNA as those displayed by wild-type cells indicates that changes in the level of ubiquitin mRNA can be compensated for by other ubiquitin-coding genes. Because previous reports have shown that UBA52 is rapidly cleaved^11^,^17^, we transfected a FLAG-UBA52-green fluorescent protein (GFP) vector into DLD-1 cells. We detected a band of approximately 34 kDa using anti-UBA52 and anti-GFP antibodies but did not detect this using anti-FLAG and anti-ubiquitin antibodies (Fig. 2B). The anti-FLAG antibody blot showed a high-molecular weight smear. These findings indicate that FLAG-UBA52–GFP is cleaved and that polyubiquitin is generated from FLAG-ubiquitin. In addition, we detected RPL40-GFP and endogenous RPL40 using an anti-UBA52 antibody. To confirm that ubiquitin was obtained from the cleavage of UBA52, we generated cleavage-resistant UBA52 (CR) expression vector, which was constructed by mutating the terminal region of ubiquitin (G75/76A)^17^. FLAG-UBA52-GFP (CR) expression vector showed decreased high-molecular weight smear as compared to FLAG-UBA52-GFP (WT) (Fig. 2C). FLAG-UBA52-GFP (CR) was detected at approximately 44 kDa by anti-FLAG antibody and anti-GFP antibody (Fig. 2C). Thus, we confirmed that ubiquitin was generated from UBA52. RPs are known to play many extra ribosomal roles^29^. For example, RPS3 regulates NF-κB signaling^30^ and RPL13a in macrophages resolves inflammation^31^. To examine the localization of UBA52, we transfected a FLAG-UBA52-GFP vector into DLD-1 cells. Microscopic analysis revealed that FLAG-GFP was expressed in both the cytosol and nucleolus. RPL40-GFP was not expressed in the nucleolus (Fig. 2D). To confirm the localization of endogenous RPL40, we lysed the cells and separated the soluble, ribosome-free cytosol (S100) from a particulate pellet containing ribosomes (P100) by ultracentrifugation^30^. RPL7a and RPS3 were expressed in the ribosomal fraction (P100) (Fig. 2E). In contrast, CDK6 and Actin were expressed in the cytosol (S100). We found that RPL40 was expressed not only in the ribosomal fraction (P100) but also in the cytosol (Fig. 2E). In addition, RPL40 expression in the ribosomal fraction (P100) gradually decreased over time after siRNA transfection. These findings showed that we could analyze ribosomal function by the siRNA approach. To better understand the roles of UBA52, we tested whether it regulates ribosomal function. A protein synthesis assay using O-propargyl-puromycin (OPP) revealed that cycloheximide (CHX) completely inhibited protein biosynthesis under these conditions. *RPS3* and *UBA52* knockdown decreased protein synthesis (Fig. 2F). To confirm the general role of UBA52, we tested Hela cells as well as DLD-1 cells (Fig. 2G). Along with DLD-1 cells, *UBA52*-deficient Hela cells showed decreased protein synthesis. Together, these data indicate that the loss of UBA52 does not regulate the total amount of ubiquitin mRNA but regulates protein synthesis functions in cells. Thus, *Uba52*-deficient lethality may be because of ribosomal dysfunction. Further investigation is required to clarify the mechanism by which UBA52 regulates embryonic development *in vivo*.

### UBA52 regulates the cell cycle

**Figure 3:**
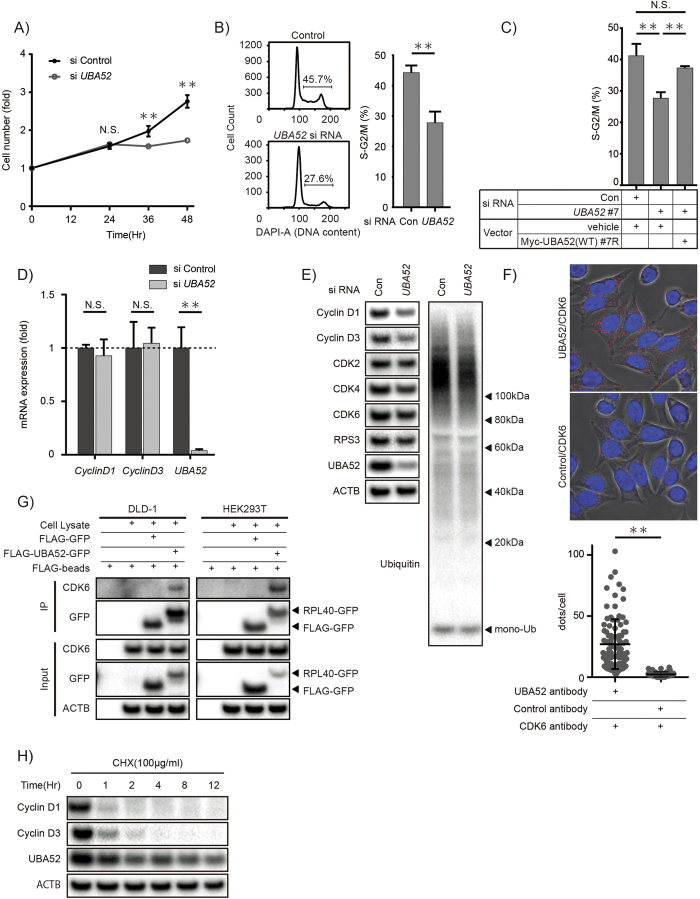
UBA52 regulates the cell cycle. (**A**) Cell viability assays in *UBA52*-deficient cells. DLD-1 cells were transfected with a *UBA52* siRNA. Then, cells were harvested for the cell viability assay at the indicated time. The fluorescent score was normalized to the level at 0 h. Data are representative of three independent experiments. **P < 0.01 (two-tailed Student’s *t*-test). (**B**) Cell-cycle analysis in *UBA52*-deficient cells. DLD-1 cells were transfected with *UBA52* siRNA. Twenty-four hours later, cells were harvested for cell-cycle analysis. Data are representative of more than three independent experiments. **P < 0.01 (one-way ANOVA followed by Tukey’s test). (**C**) Myc-UBA52 (WT) regulates the cell cycle. DLD-1 cells were transfected with Myc-UBA52 (WT) #7R and *UBA52* siRNA simultaneously. Thirty-six hours later, cells were harvested for cell-cycle analysis. Data are representative of more than three independent experiments. **P < 0.01 (one-way ANOVA followed by Tukey’s test). (**D**) Cell-cycle-related mRNA expression in *UBA52*-deficient cells. DLD-1 cells were transfected with a *UBA52* siRNA. Twenty-four hours later, cells were harvested for quantitative real-time reverse-transcription PCR and normalized to *ACTB* levels. Data are representative of three independent experiments. (**E**) Cell cycle-related protein expression in *UBA52*-deficient cells. DLD-1 cells were transfected with a *UBA52* siRNA. Twenty-four hours later, cells were harvested for immunoblotting. Data are representative of more than three independent experiments. (**F**) RPL40 co-localises with CDK6. DLD-1 cells were harvested for the *in situ* proximity ligation assay. Anti-UBA52 and anti-CDK6 antibodies were used. Data are representative of four independent experiments. **P < 0.01 (two-tailed Student’s *t*-test). (**G**) UBA52 interacts with CDK6. DLD-1 cells and HEK293T cells were transfected with FLAG-UBA52-GFP. Twenty hours later, cells were lysed and protein extracts were immunoprecipitated with GFP antibody and immunoblotted for the indicated proteins. Data are representative of more than three independent experiments in DLD-1 cells and HEK293T cells. (**H**) CHX chase experiment for the cyclin D protein. DLD-1 cells were treated with 100 μg/ml CHX and cell lysates were harvested for immunoblotting at the indicated time points. Data are representative of more than three independent experiments.

Ribosomal stress can regulate the cell cycle by p53-dependent and -independent pathways^32^,^33^. To understand the role of UBA52, we analyzed cell proliferation. We found that *UBA52*-deficient DLD-1 cells displayed decreased cell numbers and cell-cycle arrest at G1/S (Fig. 3A,B). To determine whether UBA52 expression is sufficient to regulate the cell cycle, we mutated UBA52 expression vectors to induce siRNA resistance. We found that Myc-UBA52 ameliorated the cell-cycle arrest caused by *UBA52* deficiency (Fig. 3C). Together, these findings indicate that UBA52 regulates the cell cycle. Next, to understand the mechanism underlying this, we consider that cyclin D promotes cell cycle as a main regulator^34^. We analyzed *cyclin D1* and *D3* gene expressions. There were no differences in *cyclin D1* and *D3* mRNA expressions between control and *UBA52*-deficient cells (Fig. 3D). In contrast, the levels of cyclin D1 and D3 protein expression decreased in *UBA52*-deficient cells (Fig. 3E). To examine the possibility that UBA52 regulates cyclin D expression, we analyzed cell division protein kinase 6 (CDK6), which is known to be a major partner of cyclin D and a key molecule in the regulation of G1/S phase. CDK6 expressed in the cytosol fraction (Fig. 2E). Using a proximity ligation assay, we found that endogenous RPL40 co-localized with CDK6 (Fig. 3F). To confirm physical interaction, we performed immunoprecipitation assay. Endogenous CDK6 associated with RPL40-GFP in DLD-1 cells and HEK293T cells (Fig. 3G). These data suggested that RPL40 may affect the CDK6 complex formation. Nevertheless, cyclin D1 and D3 quickly degraded after CHX treatment in DLD-1 cells (Fig. 3H). Moreover, similar observations that the normal expression of mRNAs and the decreased protein levels of cyclins D1 and D3 in *UBA52*-deficient cells were found in *Rps6*^*wt/del*^ p53^−/−^ embryos^23^. These findings indicated that decreased levels of cyclin D1 and D3 were provoked mainly by the suppression of protein synthesis in *UBA52*-deficient cells. Taken together, UBA52 modulates cyclin D1 and D3 protein expression and regulates the cell cycle.

### Ubiquitin cleaved from UBA52 forms a molecular complex with RPL40

**Figure 4:**
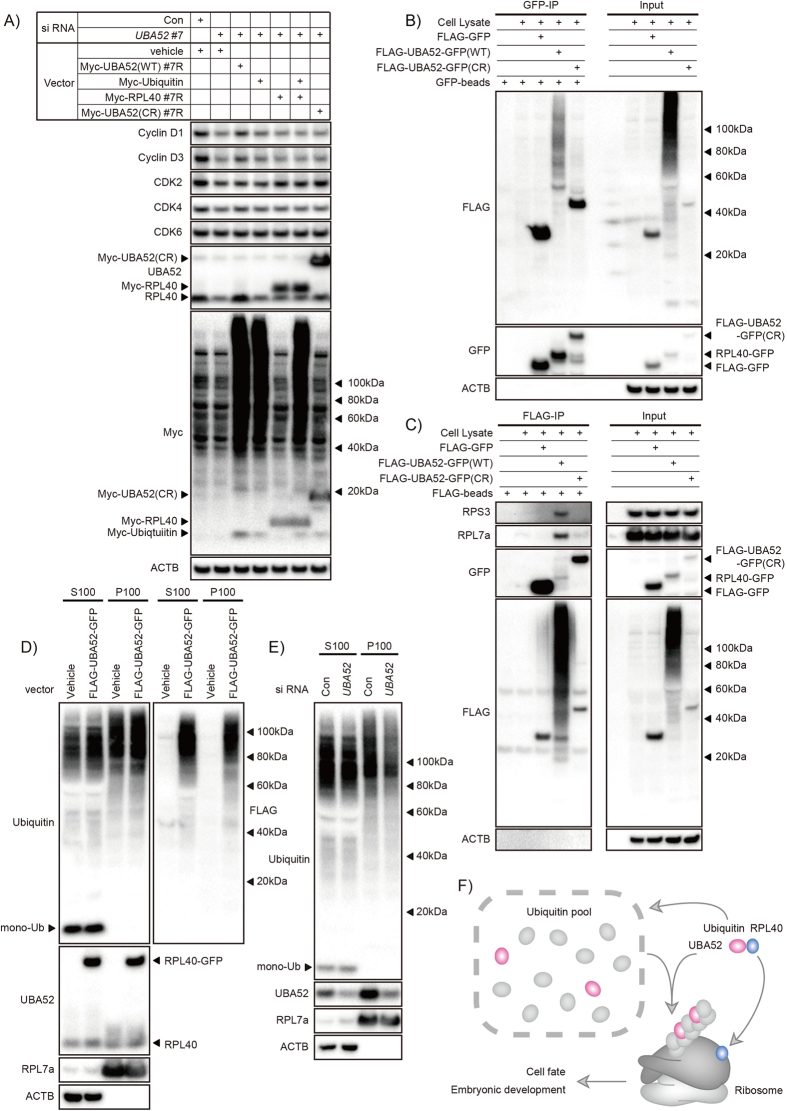
Ubiquitin cleaved from UBA52 forms a molecular complex with RPL40. (**A**) Myc-UBA52 (WT) regulates cyclin D expression. DLD-1 cells were transfected with a *UBA52* siRNA. After 6 h, DLD-1 cells were transfected with the siRNA-resistant vectors indicated. Twenty-seven hours later, cells were harvested for immunoblotting. Data are representative of more than three independent experiments. (**B**) RPL40 binds ubiquitinated molecules. DLD-1 cells were transfected with FLAG-UBA52-GFP (WT) and other vectors. Twenty-four hours later, cells were lysed and protein extracts were immunoprecipitated with GFP antibody and immunoblotted for the indicated proteins. Data are representative of more than three independent experiments. (**C**) Ubiquitin, cleaved from UBA52, forms a molecular complex with ribosome. DLD-1 cells were transfected with FLAG-UBA52-GFP (WT) and other vectors. Twenty-four hours later, cells were lysed and protein extracts were immunoprecipitated with FLAG antibody and immunoblotted for the indicated proteins. Data are representative of two independent experiments. (**D**) Subcellular fractions of FLAG-UBA52-GFP expressed DLD-1 cells. DLD-1 cells were transfected with FLAG-UBA52-GFP vector or vehicle, and 24 h later, lysed for ultracentrifugation at 10,000 × g for 1 h. S100 (cytosol) and P100 (crude ribosome pellet) fractions were immunoblotted for the indicated proteins. Data are representative of two independent experiments. (**E**) Subcellular fractions of UBA52-deficient DLD-1 cells. DLD-1 cells were transfected with *UBA52* siRNA or control siRNA, and 24 h later, ultracentrifugation was performed. Data are representative of three independent experiments. (**F**) Schematic showing that UBA52 is a dual regulator of ribosomal function. RPL40 influences the ribosomal biogenesis as a ribosomal protein, at the same time, ubiquitin cleaved from UBA52 generates ubiquitination of the ribosomal protein complex.

To understand how the ubiquitin-ribosomal hybrid gene regulates protein synthesis, we generated cleavage-resistant (CR) and siRNA resistant UBA52 expression vectors^17^. Ubiquitin, Myc-RPL40, and Myc-UBA52 (CR) overexpression in DLD-1 cells did not affect cyclin D expression, but Myc-UBA52 (WT) ameliorated the decreased cyclin D1 and D3 expressions (Fig. 4A, Supple Fig. 1). These findings indicated that RPL40 expression itself is not sufficient to regulate protein synthesis. The RPL40 cleavage mechanism and localization of ubiquitin cleaved from UBA52 may influence the function of UBA52. Thus, we tested whether UBA52 and UBA52 (CR) formed a molecular complex. GFP immunoprecipitation revealed that RPL40 bound molecules that had been ubiquitinated using ubiquitin generated from UBA52 cleavage (Fig. 4B). Furthermore, FLAG immunoprecipitation revealed that ubiquitin cleaved from UBA52 ubiquitinated the ribosomal complex (Fig. 4C). These findings indicated that UBA52 supplies ubiquitin to the ribosomal complex. To confirm whether UBA52 regulates the ribosomal protein complex, we tested the ribosomal fraction. FLAG-UBA52-GFP transfected cell showed that both FLAG-ubiquitin from UBA52 and RPL40-GFP expressed in the ribosomal fraction (Fig. 4D). In addition, *UBA52*-deficient cell showed reduced ubiquitin smear in the ribosomal fraction (Fig. 4E). Taken together, these data suggested that RPL40 formed a ribosomal protein complex with ubiquitin cleaved from UBA52 (Fig. 4F). Recent studies have shown that the regulation of ubiquitin-dependent translation is an important feature of cell-fate determination^35^. Regulatory 40 S ribosomal ubiquitination events play a critical role in protein biogenesis^36^. Our findings demonstrated that ubiquitin, RPL40, UBA52 (CR), and a mixture of ubiquitin and RPL40 could not ameliorate cyclin D1 and D3 expressions (Fig. 4A). However, full-length UBA52 abolished the cell-cycle-related *UBA52*-deficient phenotype. These findings indicate that the RPL40 cleavage is critical for the functions of UBA52. Thus, we hypothesize that UBA52 regulates ribosomal function by two steps. RPL40 influences the ribosomal biogenesis as a ribosomal protein, at the same time, ubiquitin cleaved from UBA52 generates ubiquitination of the ribosomal protein complex. These data reveal a novel mechanism that ubiquitin regulates translation by the ubiquitin–ribosomal hybrid gene. Future studies will be needed to identify enzymes that cleave ubiquitin from UBA52 under physiological conditions. Moreover, it is critical to analyze UBA52’s physiological functions in cell type-specific contexts.

In conclusion, the generation of mice lacking *Uba52* has allowed us to unveil the physiological function of the ubiquitin hybrid gene *Uba52*. In particular, we have discovered that UBA52 regulates cell fate and embryonic development. RPL40, the ribosomal domain of UBA52, generated a ribosomal protein complex, at the same time, the UBA52 ubiquitin domain simultaneously generated ubiquitination of the ribosomal complex. Thus, UBA52 is a dual regulator of ribosomal protein complex. These findings provide unique molecular insights into ubiquitin-related protein synthesis and embryonic development.

## Methods

### Mice

*Uba52*^*tm1a(EUCOMM)Wtsi*^ embryonic stem cells were purchased from the European Conditional Mouse Mutagenesis Program (EUCOMM) and microinjected into the blastocysts of an albino C57BL6 strain. The chimeric mice were backcrossed with the same strain of albino C57BL6 mice to generate heterozygous *Uba52* mutant mice. All animal experiments were approved by the Institutional Animal Care and Use Committee of the Tokyo Medical and Dental University. Experiments were performed in compliance with Tokyo Medical and Dental University’s Animal Facility regulations. Genotypes were initially confirmed by Southern blotting using embryonic stem cells. In addition, genotypes were confirmed by PCR using DNA derived from the tail and the following primers: Primer4, F 5′-CTGCAGAGGGAGTTCAGGG-3′ and R 5′-GTTTGGTAAGTAGGGGCAGC-3′; Primer5, F 5′-FACAACCATGGAAGATCCCGT-3′ and R 5′-CCGTTGCACCACAGATGAAA-3′ and Primer6, F 5′-AGGAAGGAGTTGTGGCCAACCTGG-3′ and R 5′-TGAACTGATGGCGAGCTCAGACC-3′. Also, the following primers were used for long-range PCR: Primer1, F 5′-TCCAGACAGAACGACTATTCTCGC-3′ and R 5′-AACTGAAGGATCGGACAGCA-3′; Primer2, F 5′-ACAACCATGGAAGATCCCGT-3′ and R 5′-AACTGAAGGATCGGACAGCA-3′ and Primer3, F 5′-TCCAGACAGAACGACTATTCTCGC-3′ and R 5′-CCGTTGCACCACAGATGAAA-3′.

### Southern blotting

A DNA template was extracted from embryonic stem cells purchased from EUCOMM. Probes were set to correspond with a sequence of 505 base pairs (bp) in the region containing exon 1 (forward primer, 5′-GCTCGGCCTAGGATTCATTT-3′; reverse primer, 5′-CGCCTCGTTGAAGAGAAAGA-3′). The DNA template was digested using *Eco*RI and *Pst*I. The digested DNA was electrophoresed in a 1–1.5% agarose gel and transferred to a positively charged nylon membrane (Hybond-N+, RPN119B; GE Healthcare, Little Chalfont, Buckinghamshire, UK). DIG Easy Hyb (1603558; Roche Diagnostics, Basel, Switzerland) was used for hybridization, and ready-to-use disodium 3-{4-methoxyspiro[1,2-dioxetane-3,2′-(5′-chloro)tricycle(3.3.1.1^3^,^7^)decan]-4-yl}phenyl phosphate (11755633001; Roche Diagnostics) was used for detection, as described in the manufacturer’s protocol.

### Cell culture and reagents

The human colorectal cancer cell line DLD-1 was cultured in Roswell Park Memorial Institute-1640 medium (R8758; Sigma-Aldrich Corp., St. Louis, MO, USA) with 10% fetal bovine serum (FBS; S1820; Biowest SAS, Nuaillé, France) and 1% penicillin/streptomycin (26253-84; Nacalai Tesque, Inc., Kyoto, Japan) at 37 °C. HEK293T cells and Hela cells were cultured in Dulbecco’s Modified Eagle’s Medium (high glucose) (D5796; Sigma-Aldrich Corp., St. Louis, MO, USA) with 10% FBS and 1% penicillin/streptomycin at 37 °C. Cells were transiently transfected with siRNAs using Lipofectamine^®^ RNAiMAX™ (13778150; Invitrogen, Carlsbad, CA, USA), as indicated by the manufacturer. DLD-1 cells were transiently transfected with plasmid vectors using Lipofectamine^®^ LTX & Plus™ (15338-100; Invitrogen) or Lipofectamine^®^ 3000 (L3000015; Invitrogen). DLD-1 cells were co-transfected with an siRNA and a plasmid vector for the cell-cycle assay using Lipofectamine^®^ 3000 (L3000075; Invitrogen), as indicated by the manufacturer. DLD-1 cells were treated with CHX (06741-91; Nacalai Tesque, Inc.).

### Plasmids and short interfering RNA oligonucleotides

Human UBA52 cDNA was cloned from the HeLa cell line. Myc-ubiquitin was constructed by TAA (stop codon) insertion at 229–231 bp in *UBA52* cDNA using the PrimeSTAR mutagenesis basal kit (R046A; Takara Bio Inc., Shiga, Japan). Myc-RPL40 was constructed by the deletion of ubiquitin at 4–228 bp. To make CR UBA52, alanine scanning was performed every two bases in the region connecting ubiquitin and RPL40. Finally, the UBA52 (CR) vectors were constructed by mutating the connecting region of ubiquitin and RPL40 (223–234 bp; ggtggcattatt) to gctgccattatt (G75/76A). ON-TARGETplus SMARTpool siRNA oligonucleotides specific for human *UBA52*, mouse *UBA52*, human *RPS3*, and a non-targeting pool siRNA were purchased from GE Dharmacon (Lafayette, CO, USA). The same human *RPS3* siRNA sequence as that of the GE Dharmacon SMARTpool siRNAs was purchased from Hokkaido System Science Co., Ltd. Individual human *UBA52* (J-011794-07, GCUGUCAACUGCCGCAAGA; UBA52 #7) (J-011794-05, CCUGCGAGGUGGCAUUAUU; UBA52 #5), siRNA-resistant Myc-UBA52 vectors [Myc-UBA52 (WT) #7R, Myc-UBA52 (CR) #7R, and Myc-RPL40 #7R] were constructed by mutation of the RPL40 region (319–337 bp) to GCTGTCAACTGTAGGAAGA, which had no impact on the encoded protein sequence. Myc-UBA52(WT) #5R vector was constructed by mutation of the connecting region of Ubiquitin and RPL40 (216–234 bp) to CTTAAGGGGTGGCATTATT, which had no impact on the encoded protein sequence too.

### Flow cytometry

Cells were washed with phosphate-buffered saline (PBS) and dissociated using trypsin–ethylenediaminetetraacetic acid. Cells were then washed and resuspended in PBS. Iced 80% ethanol was added to a final concentration of 70%. The resuspended cells were incubated on ice for 30 min. Cells were then washed twice in cold PBS and stained with 4′,6-diamidino-2-phenylindole (DAPI)/TX-100 solution [0.1% Triton X-100, 1 μg/ml DAPI (D3571; Invitrogen) in PBS] for 30 min at room temperature. The constituent DNA amount was measured using a FACSCanto™ II flow cytometer (BD Biosciences) and cell-cycle status was analyzed using FlowJo Enterprise software (version 7.6.5; FlowJo, LLC, Ashland, OR, USA).

### Quantitative real-time (RT) PCR

For *in vitro* assays, total RNA was isolated using the RNeasy^®^ Mini Kit (Qiagen NV, Venlo, Limburg, The Netherlands). *In vivo* assays used 10–20 mg of tissue homogenized using PRECELLYS^®^ 24 and CK14 ceramic beads in a tube (KT03961-1-003.2; PRECELLYS, Montigny-le-Bretonneux, France). Total RNA was isolated using the MAgNA Pure 96 System (Roche Diagnostics) or RNeasy^®^ Mini Kit. cDNA was synthesized using the QuantiTect Reverse Transcription Kit (205313; Qiagen NV) or Transcriptor Universal cDNA Master Kit (5893151; Roche Diagnostics). Quantitative PCR was performed using the QuantiTect SYBR Green PCR Kit (204145; Qiagen NV) and the StepOnePlus™ RT-PCR System (Applied Biosystems, Foster City, CA, USA). Gene expression was normalized to the expression of the housekeeping gene *ACTB*. The contributions of four ubiquitin-coding genes to total ubiquitin levels are shown after normalization by the number of ubiquitin moieties that each ubiquitin transcript generates as follows^14^,^15^,^16^:


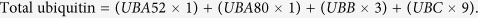


Gene expression was determined using the following primers: human *UBA52*, 5′-GCGTCCCAAGAAGAAGGTCA-3′ and 5′-ACCAATTGCTGCTCCAGTCA-3′; human *UBA80*, 5′-ACTGTTTCAACAAACCAGAAGACA-3′ and 5′-AGGAAATGGTGTGACCATCAA-3′; human *UBB*, 5′-CCTGAGGGGTGGCTGTTA-3′ and 5′-TTAACATTTTGAACAGGTTCAGCTA-3′; human *UBC* 5′-CCACTCTGCACTTGGTCCTG-3′ and 5′-GGAATGCAACAACTTTATTGAAAGG-3′; human *Cyclin D1* 5′-AGCTGTGCATCTACACCGAC-3′ and 5′-GAAATCGTGCGGGGTCATTG-3′; human *Cyclin D3* 5′-TGCACATGATTTCCTGGCCT-3′ and 5′-CTGTAGCACAGAGGGCCAAA-3′; human *RPS3* 5′-GAGTCTCTGCGTTACAAACTCC-3′ and 5′-TTTCCCAGACACCACAACCT-3′; human *ACTB* 5′-GGATGCAGAAGGAGATCACTG-3′ and 5′-CGATCCACACGGAGTACTTG-3′; mouse *Uba52* 5′-GTCAGCTTGCCCAGAAGTAC-3′ and 5′-ACTTCTTCTTGCGGCAGTTG-3′; mouse *Actb* 5′-TTGGGTATGGAATCCTGTGG-3′ and 5′-GTACT TGCGCTCAGG AGGAG-3′.

### Microscopic analysis

Cells were washed with PBS and fixed in 4% paraformaldehyde for 15 min at 4 °C. Fixed cells were washed with PBS twice and dehydrated in 100% methanol for 10 min at 4 °C. The nucleolus was stained using VECTASHIELD^®^ Mounting Medium with DAPI (H-1200; Vector Laboratories, Inc., Burlingame, CA, USA). Images were acquired using a confocal laser microscope (FV10i; Olympus Corp., Tokyo, Japan) with a 60× oil immersion objective lens.

### Immunoblotting

Cells were incubated in lysis buffer {either 20 mM 4-(2-hydroxyethyl)-1-piperazineethanesulfonic acid (HEPES; pH 7.5), 150 mM NaCl, 0.5% Triton X-100, 0.5% 3-[(3-cholamidopropyl)dimethylammonio]-1-propanesulfonate (CHAPS), and 10% glycerol or 20 mM Tris–HCl (pH 7.5), 150 mM NaCl, 0.2% NP-40, and 10% glycerol} with added Halt protease and phosphatase inhibitor cocktail (1861280; Pierce Biotechnology, Rockford, IL, USA), 2 mM *N*-ethylmaleimide (15512-24; Nacalai Tesque Inc.), and 10 μM MG-132 on ice for 20 min and centrifuged at 14,000 × *g* for 20 min. For FLAG or GFP immunoprecipitation, cell lysates were incubated with Anti-DDDDK-tag mAb-Magnetic Beads (M185-9; MBL International Corporation, Nagoya, Japan) or Anti-GFPmAb-Magnetic Beads (D153-9; MBL International Corporation). Cell lysates were incubated with the pre-coupled beads for 3 h in GFP-IP or 1 h in FLAG-IP at 4 °C and then washed twice with lysis buffer with added NaCl (final concentration, 450 mM). Samples were resolved on NuPage precast 4–12% Bis-Tris gels (NP0323; Invitrogen) and transferred to polyvinylidene difluoride membranes. The following antibodies and reagents were used for immunoprecipitation and immunoblotting studies: anti-ACTB (A5441; Sigma-Aldrich Corp.); anti-FLAG (F7425; Sigma-Aldrich Corp.); anti-Myc (A-14; sc-789; Santa Cruz Biotechnology, Inc., Dallas, TX, USA); anti-GFP (A11122; Life Technologies, Carlsbad, CA, USA); anti-UBA52 (EPR4546, ab109227, Abcam, Cambridge, UK; and EPR4547, ab109230, Abcam); anti-ubiquitin (P4D1; sc-8017; Santa Cruz Biotechnology, Inc.); anti-RPS3 (D50G7; 9538; Cell Signaling Technology, Inc.); anti-RPL7a (E109; 2415; Cell Signaling Technology, Inc.); anti-cyclin D1 (M-20; sc-718; Santa Cruz Biotechnology, Inc.); anti-cyclin D3 (DCS22; 2936; Cell Signaling Technology, Inc.); anti-CDK2 (78B2; 2546; Cell Signaling Technology, Inc.); anti-CDK4 (C-22; sc-260; Santa Cruz Biotechnology, Inc.); and anti-CDK6 (C-21; sc-177; Santa Cruz Biotechnology, Inc.).

### Ultracentrifugation

Cells were incubated in lysis buffer without glycerol [20 mM HEPES (pH 7.5), 150 mM NaCl, 0.5% TritonX-100, and 0.5% CHAPS] with added Halt protease and phosphatase inhibitor cocktail on ice for 20 min and centrifuged at 14,000 × *g* for 20 min to remove the nuclear fraction. Then, the supernatant was ultracentrifuged at 4 °C and 100,000 × *g* for 1 h. The supernatant was preserved as the S100 fraction. The pellet was washed with PBS and ultracentrifuged again at 4 °C and 100,000 × *g* for 20 min. The supernatant was discarded and the pellet, which was the P100 fraction, was refused in lysis buffer using an ultrasonic homogenizer.

### Protein synthesis assay

To analyze protein synthesis in cells, a Click-iT^®^ Plus OPP Protein Synthesis Assay Kit (Alexa Fluor^®^ 488; C104567570; Molecular Probes, Eugene, OR, USA) was used in accordance with the manufacturer’s protocol. The GloMax^®^ Discover System (Promega Corp.) was used to scan the plates.

### Cell viability assay

CellTiter-Glo Luminescent Cell Viability Assay (G7570; Promega Corp.) was used to count viable cells in wells, as indicated by the manufacturer.

### *In situ* proximity ligation assay

To detect protein interactions in cells, a Duolink^®^ PLA *in-situ* kit (92101; Sigma–Aldrich Corp.) was used according to the manufacturer’s instructions. The primary antibodies were as follows: a rabbit anti-UBA52 antibody (EPR4547; ab109230; Abcam) and a mouse anti-CDK6 antibody (ab54576; Abcam). The primary antibody rabbit anti-Immunoglobulin G was used as a control (ab125938; Abcam). Images were acquired with a confocal laser microscope using a ×60 oil immersion objective lens. Dots within cells were counted using Duolink ImageTool (OLINK Bioscience, Uppsala, Sweden). Cells were only counted when the whole cell was in the field of vision.

### Statistical Analysis

Data were analyzed using the two-tailed unpaired Student t-test or one-way ANOVA followed by Tukey’s test using Prism software (GraphPad Software, Inc.).

## Additional Information

**How to cite this article**: Kobayashi, M. *et al*. The ubiquitin hybrid gene *UBA52* regulates ubiquitination of ribosome and sustains embryonic development. *Sci. Rep*. **6**, 36780; doi: 10.1038/srep36780 (2016).

**Publisher’s note**: Springer Nature remains neutral with regard to jurisdictional claims in published maps and institutional affiliations.

## Supplementary Material

Supplementary Information

Supplementary Figures
