# Supplementary material for: The ubiquitin hybrid gene UBA52 regulates ubiquitination of ribosome and sustains embryonic development
Source: Sci Rep. 2016 Nov 10;6:36780. doi: 10.1038/srep36780 (PMC5103194; doi:10.1038/srep36780)
Supplement: Supplementary Information [file srep36780-s1.pdf]

The ubiquitin hybrid gene *UBA52* regulates ubiquitination of ribosome and sustains embryonic development

Masanori Kobayashi[a], Shigeru Oshima[a]*, Chiaki Maeyashiki[a], Yoichi Nibe[a], Kana Otsubo[a], Yu Matsuzawa[a], Yasuhiro Nemoto[a], Takashi Nagaishi[a], Ryuichi Okamoto[a,b], Kiichiro Tsuchiya[a], Tetsuya Nakamura[c], and Mamoru Watanabe[a]

[a]Department of Gastroenterology and Hepatology, Graduate School, Tokyo Medical and Dental University (TMDU), Tokyo 113-8510, Japan

[b]Center for Stem Cell and Regenerative Medicine, Tokyo Medical and Dental University (TMDU), Tokyo 113-8510, Japan

[c]Department of Advanced Therapeutics for GI Diseases, Tokyo Medical and Dental University (TMDU), Tokyo 113-8510, Japan

*Corresponding author: Shigeru Oshima, M.D., Ph.D., E-mail: soshima.gast@tmd.ac.jp

Department of Gastroenterology and Hepatology

Tokyo Medical and Dental University (TMDU)

1-5-45, Yushima, Bunkyo-ku, Tokyo 113-8519, Japan

Phone No.: +81-3-5803-5877

Fax No.: +81-3-5803-0268

S1)

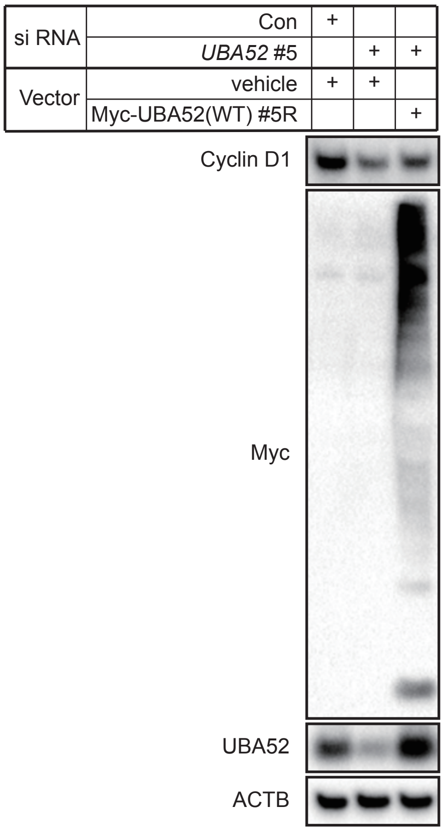

<u>Supplementary Figure 1.</u>

Myc-UBA52 (WT) regulates cyclin D expression. DLD-1 cells were transfected with a *UBA52* #5 siRNA. After 6 h, DLD-1 cells were transfected with the siRNA-resistant vectors #5R indicated. Twenty-seven hours later, cells were harvested for immunoblotting. Data are representative of more than three independent experiments. Note that *UBA52*-deficient cell by #5 siRNA also displayed the decreased Cyclin D1 expression, and that Myc-UBA52 (WT) #5R ameliorated the decreased expression of Cyclin D1
