# Supplementary material for: The ubiquitin hybrid gene UBA52 regulates ubiquitination of ribosome and sustains embryonic development
Source: Sci Rep. 2016 Nov 10;6:36780. doi: 10.1038/srep36780 (PMC5103194; doi:10.1038/srep36780)
Supplement: Supplementary Figures [file srep36780-s2.pdf]

The ubiquitin hybrid gene *UBA52* regulates ubiquitination of ribosome and sustains embryonic development

Masanori Kobayashi[a], Shigeru Oshima[a]*, Chiaki Maeyashiki[a], Yoichi Nibe[a], Kana Otsubo[a], Yu Matsuzawa[a], Yasuhiro Nemoto[a], Takashi Nagaishi[a], Ryuichi Okamoto[a,b], Kiichiro Tsuchiya[a], Tetsuya Nakamura[c], and Mamoru Watanabe[a]

[a]Department of Gastroenterology and Hepatology, Graduate School, Tokyo Medical and Dental University (TMDU), Tokyo 113-8510, Japan

[b]Center for Stem Cell and Regenerative Medicine, Tokyo Medical and Dental University (TMDU), Tokyo 113-8510, Japan

[c]Department of Advanced Therapeutics for GI Diseases, Tokyo Medical and Dental University (TMDU), Tokyo 113-8510, Japan

*Corresponding author: Shigeru Oshima, M.D., Ph.D., E-mail: soshima.gast@tmd.ac.jp

Department of Gastroenterology and Hepatology

Tokyo Medical and Dental University (TMDU)

1-5-45, Yushima, Bunkyo-ku, Tokyo 113-8519, Japan

Phone No.: +81-3-5803-5877

Fax No.: +81-3-5803-0268

1B)

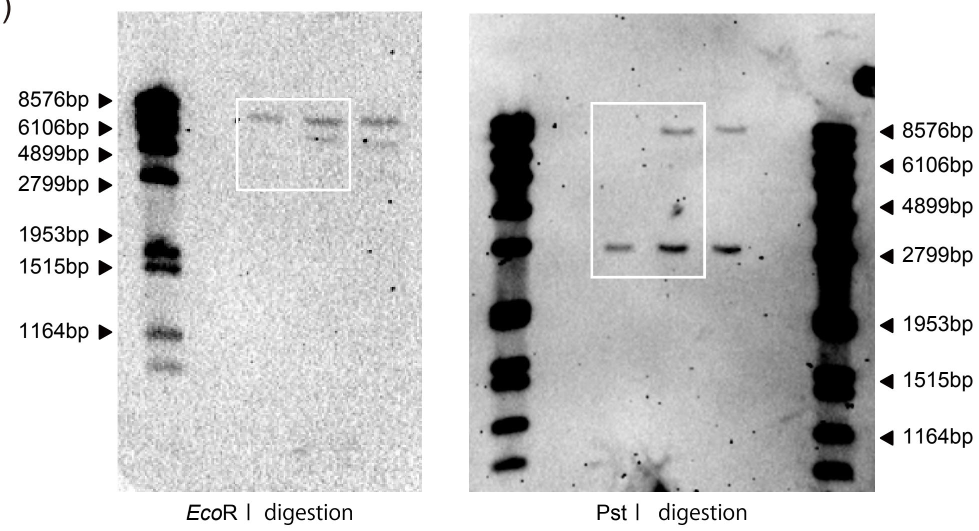
8576bp
6106bp
4899bp
2799bp
1953bp
1515bp
1164bp
8576bp
6106bp
4899bp
2799bp
1953bp
1515bp
1164bp
*Eco*R I digestion
Pst I digestion

1C)

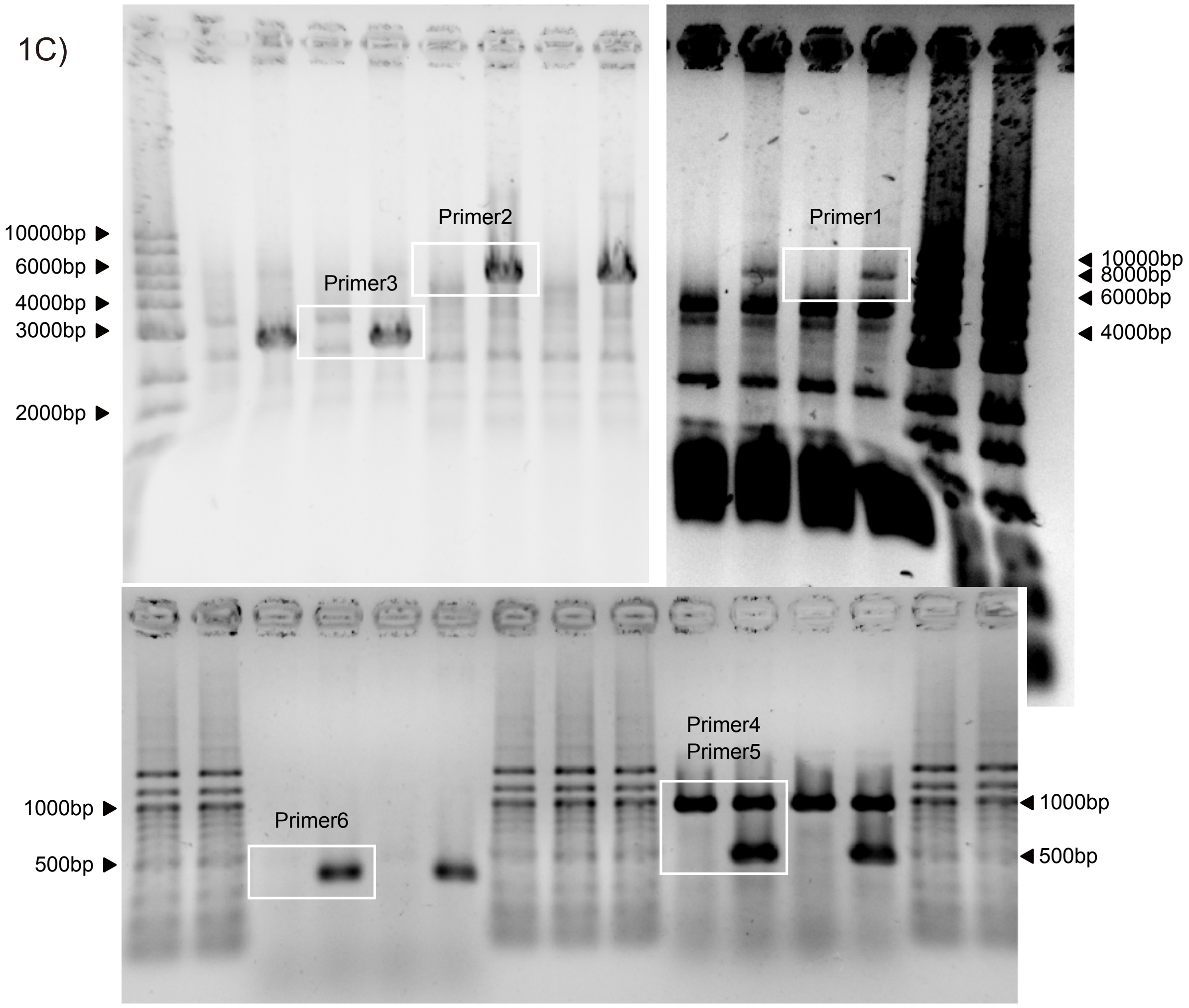
10000bp
6000bp
4000bp
3000bp
2000bp
Primer3
Primer2
Primer1
10000bp
8000bp
6000bp
4000bp
1000bp
500bp
Primer6
Primer4
Primer5
1000bp
500bp

2B)

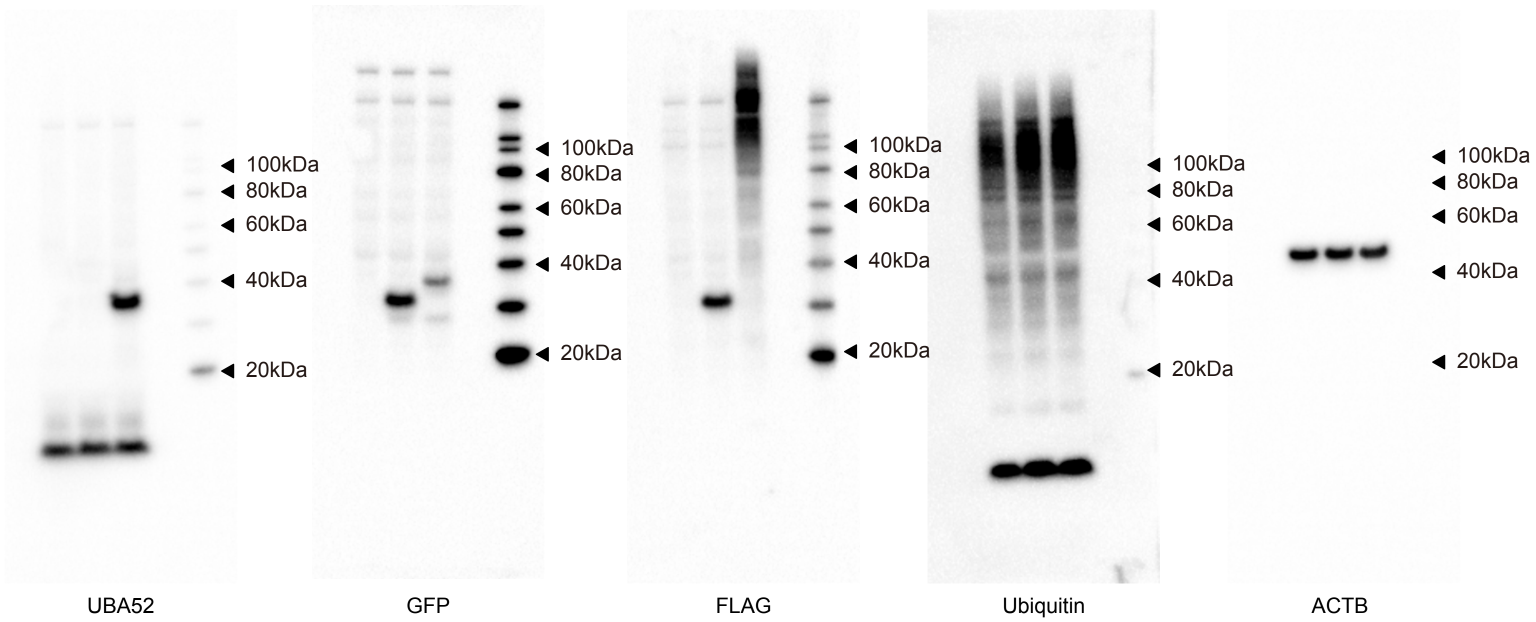

100kDa
80kDa
60kDa
40kDa
20kDa
UBA52
GFP
FLAG
Ubiquitin
ACTB

2C)

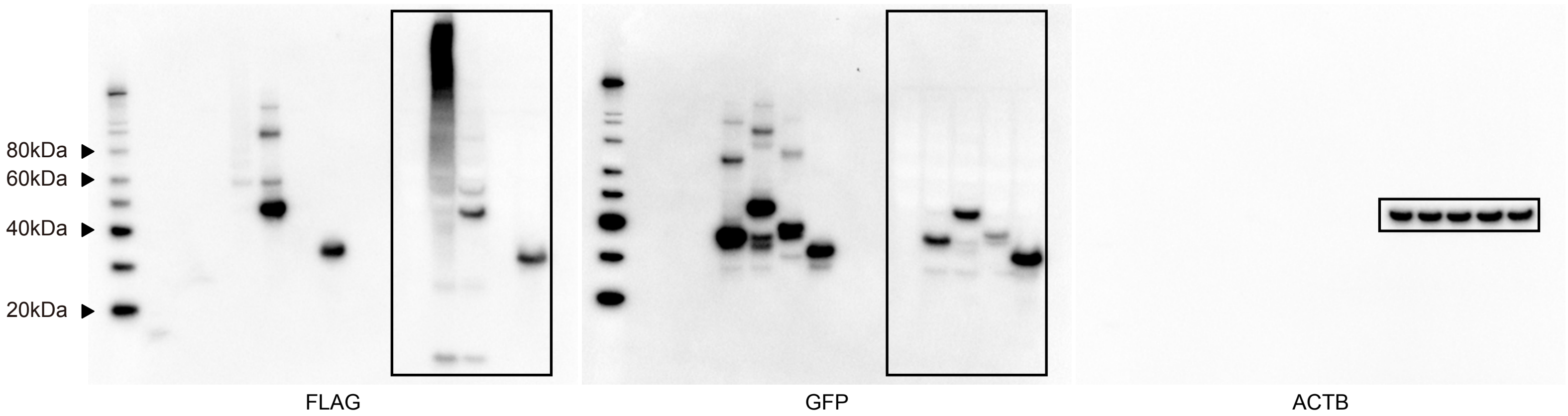

80kDa
60kDa
40kDa
20kDa
FLAG
GFP
ACTB

2E)

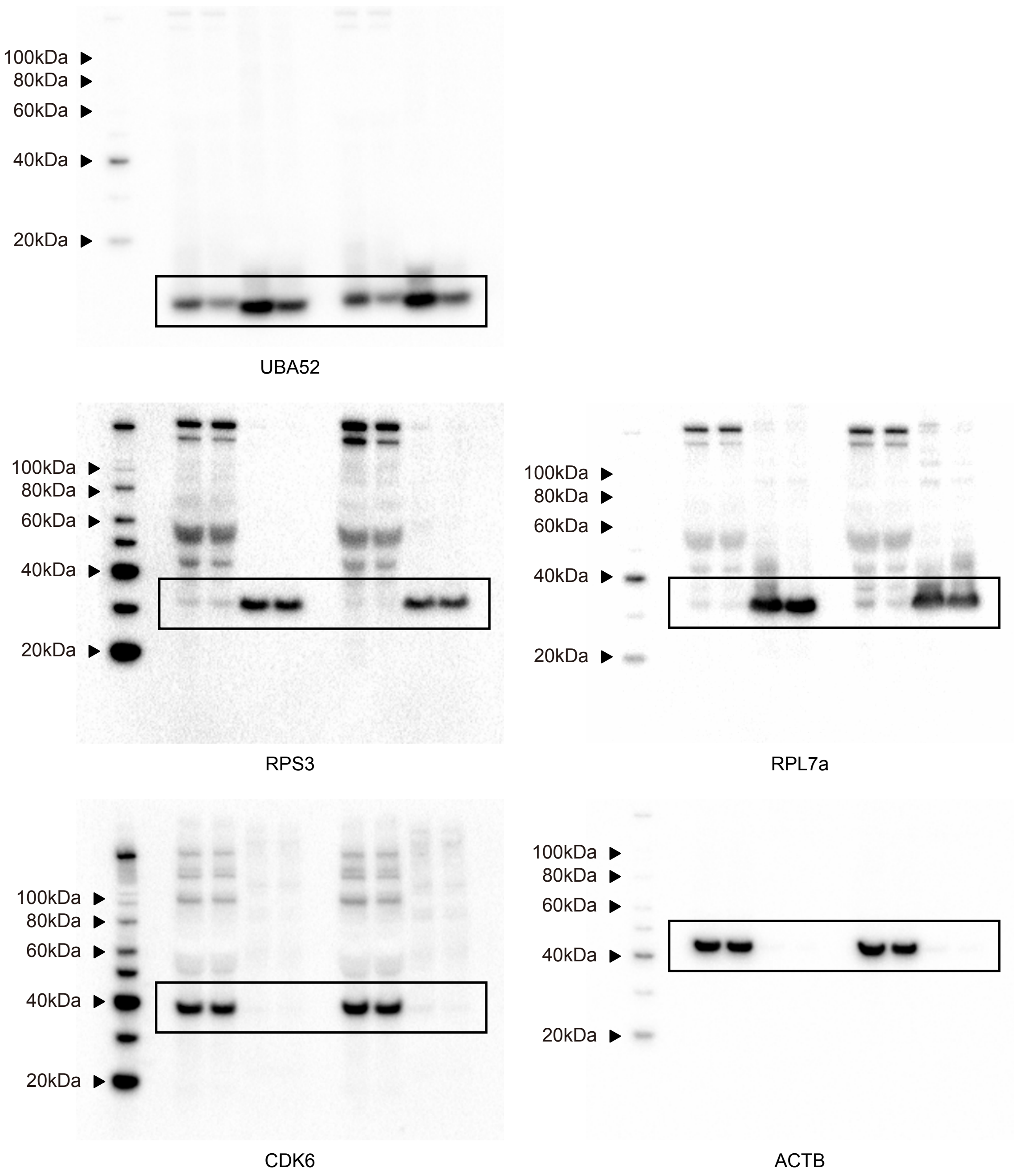

3E)

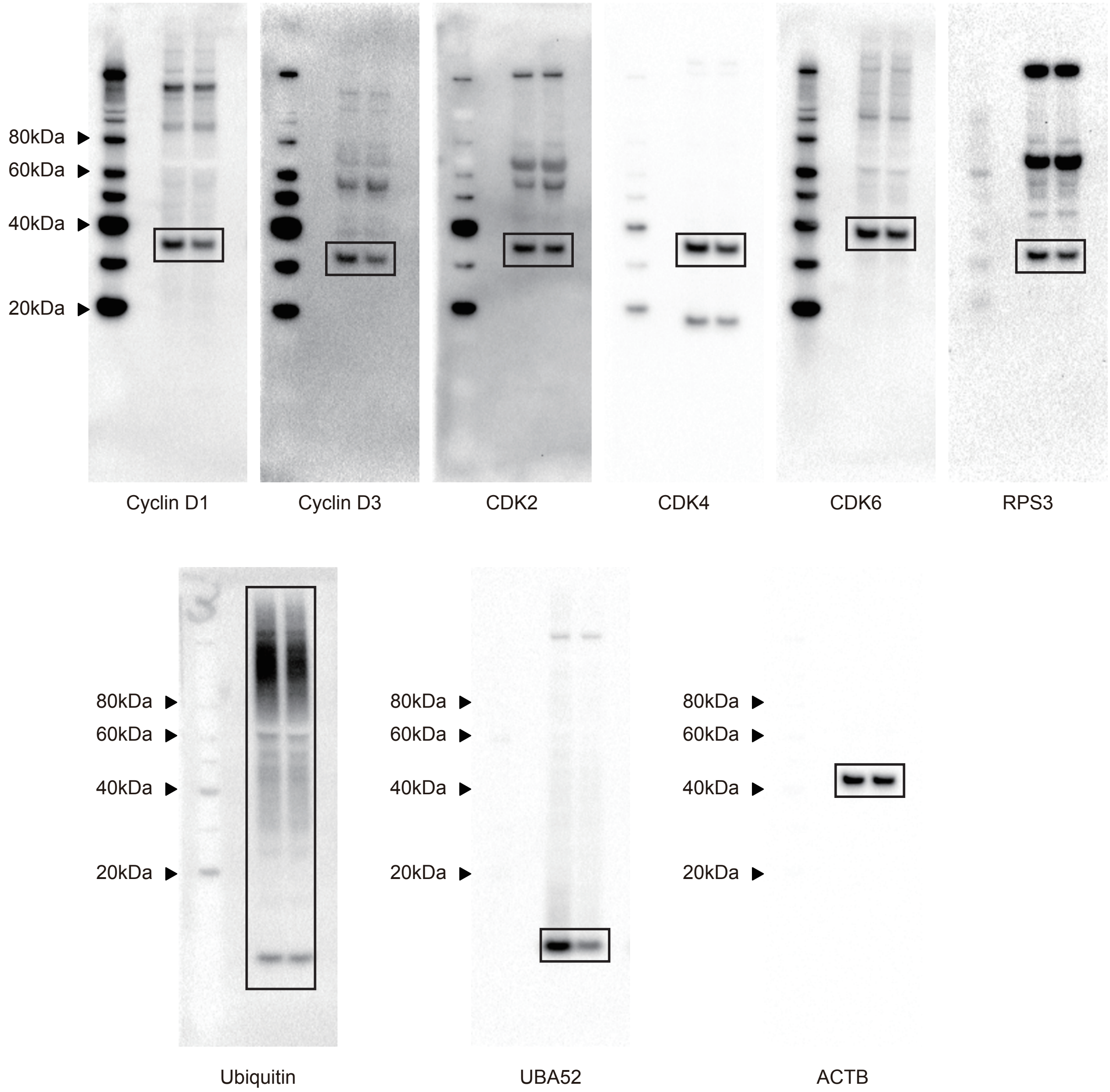
80kDa
60kDa
40kDa
20kDa
Cyclin D1
Cyclin D3
CDK2
CDK4
CDK6
RPS3
Ubiquitin
UBA52
ACTB

3G: DLD-1)

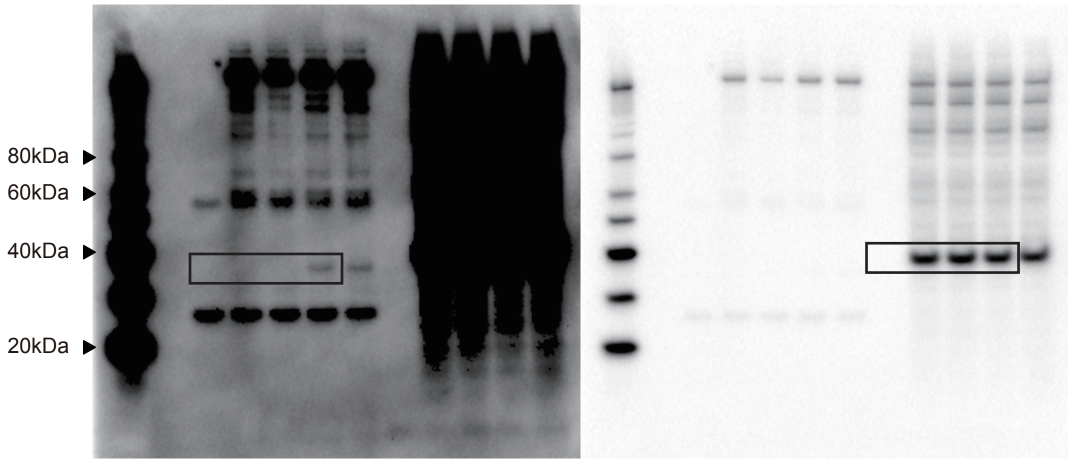

CDK6

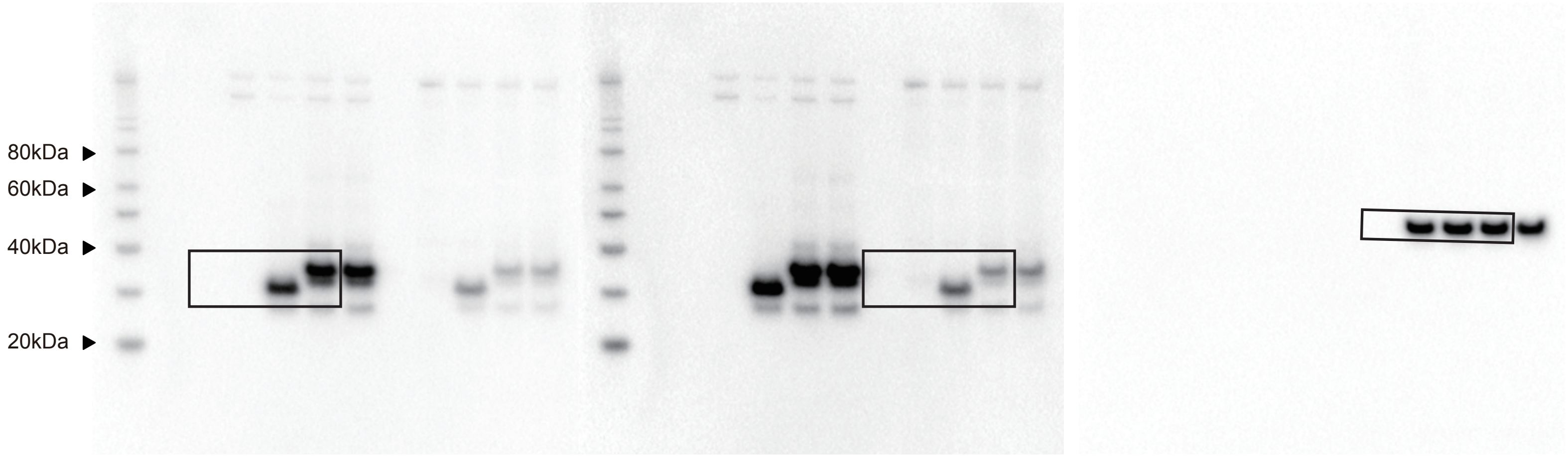

GFP

ACTB

3G: HEK293T)

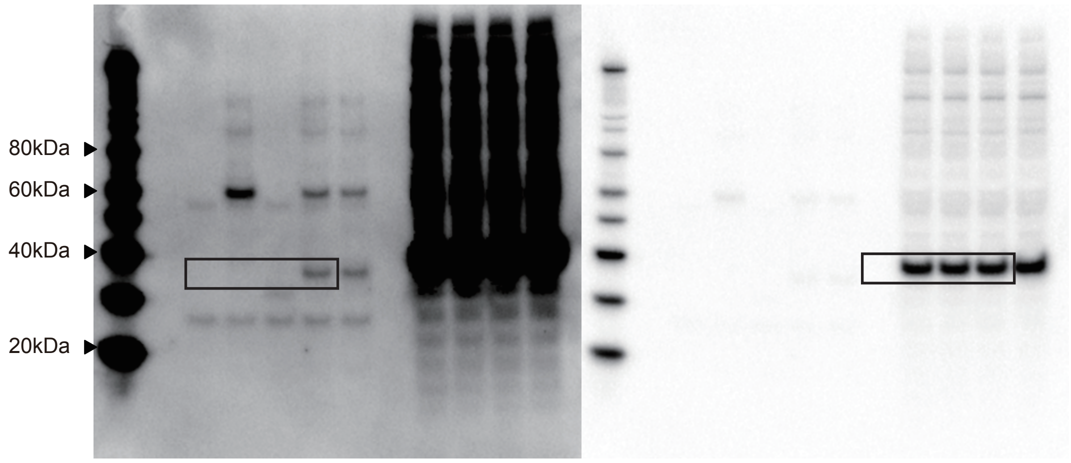

CDK6

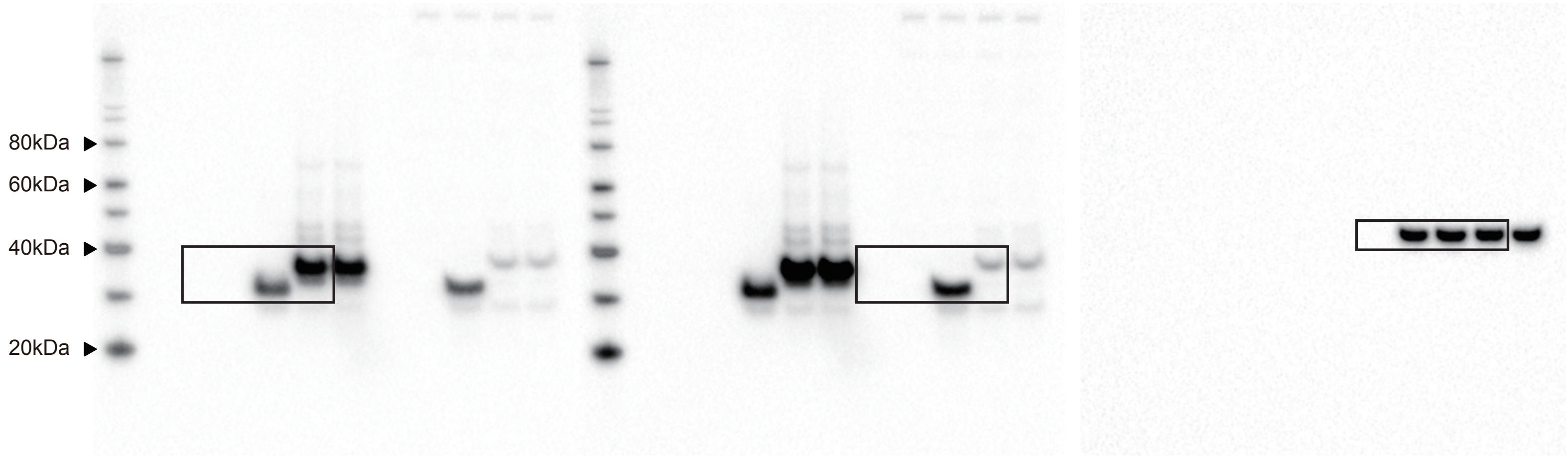

GFP

ACTB

3H)

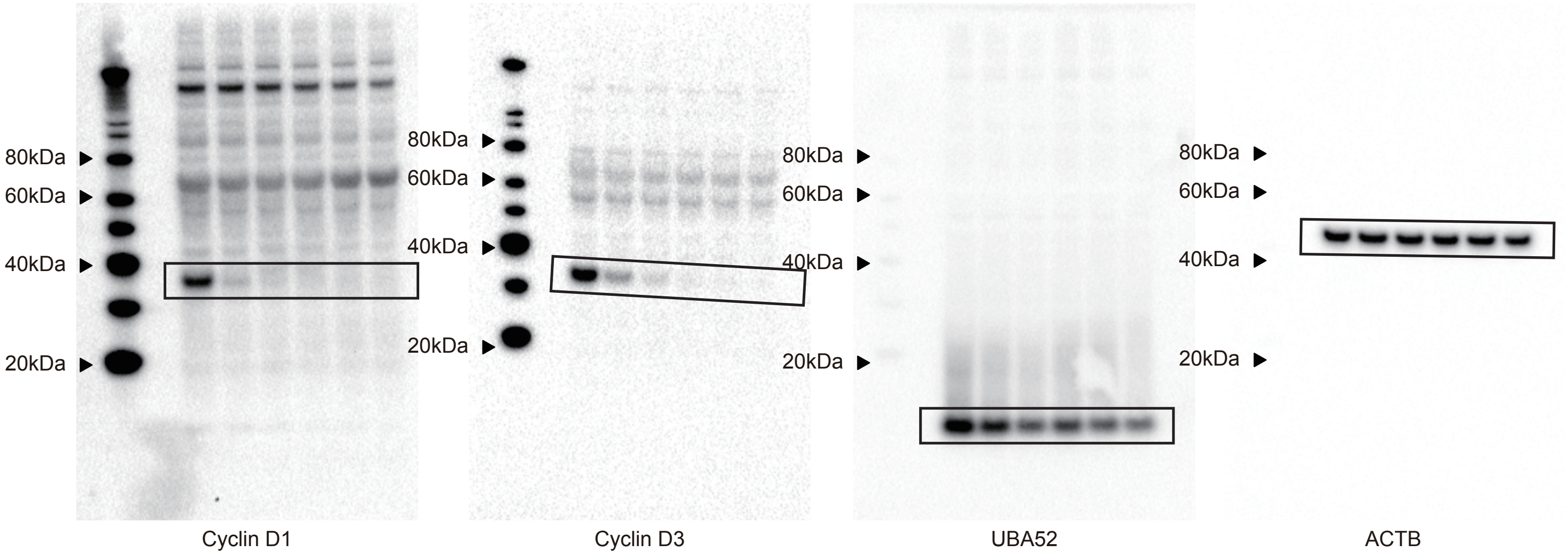
80kDa
60kDa
40kDa
20kDa
Cyclin D1
80kDa
60kDa
40kDa
20kDa
Cyclin D3
80kDa
60kDa
40kDa
20kDa
UBA52
80kDa
60kDa
40kDa
20kDa
ACTB

4A)

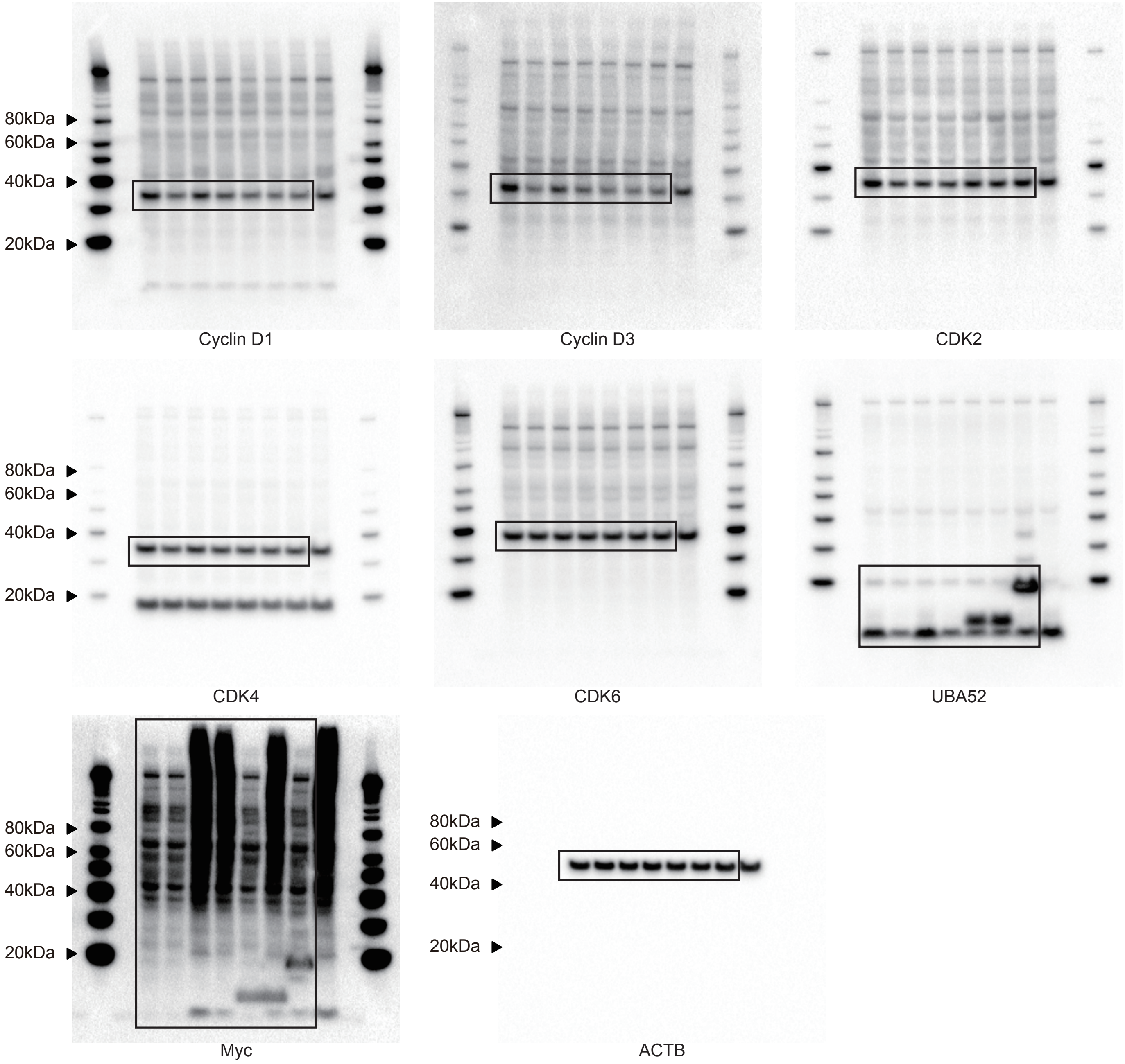

80kDa
60kDa
40kDa
20kDa
Cyclin D1
Cyclin D3
CDK2
CDK4
CDK6
UBA52
Myc
ACTB

4B)

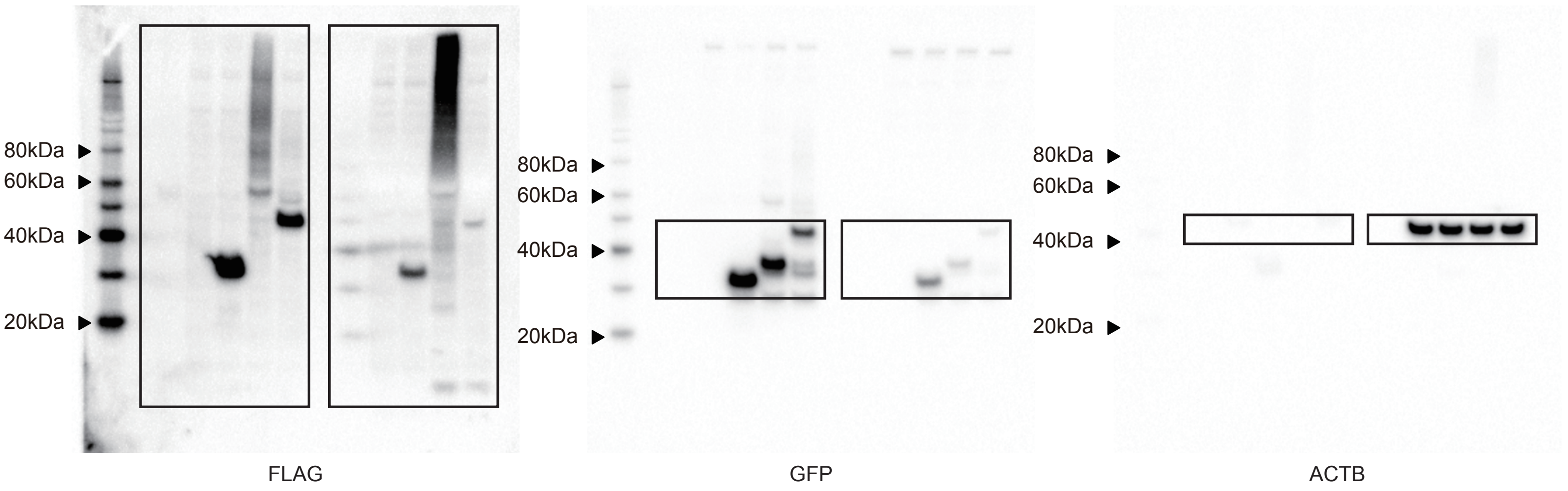
80kDa
60kDa
40kDa
20kDa
FLAG
80kDa
60kDa
40kDa
20kDa
GFP
80kDa
60kDa
40kDa
20kDa
ACTB

4C)

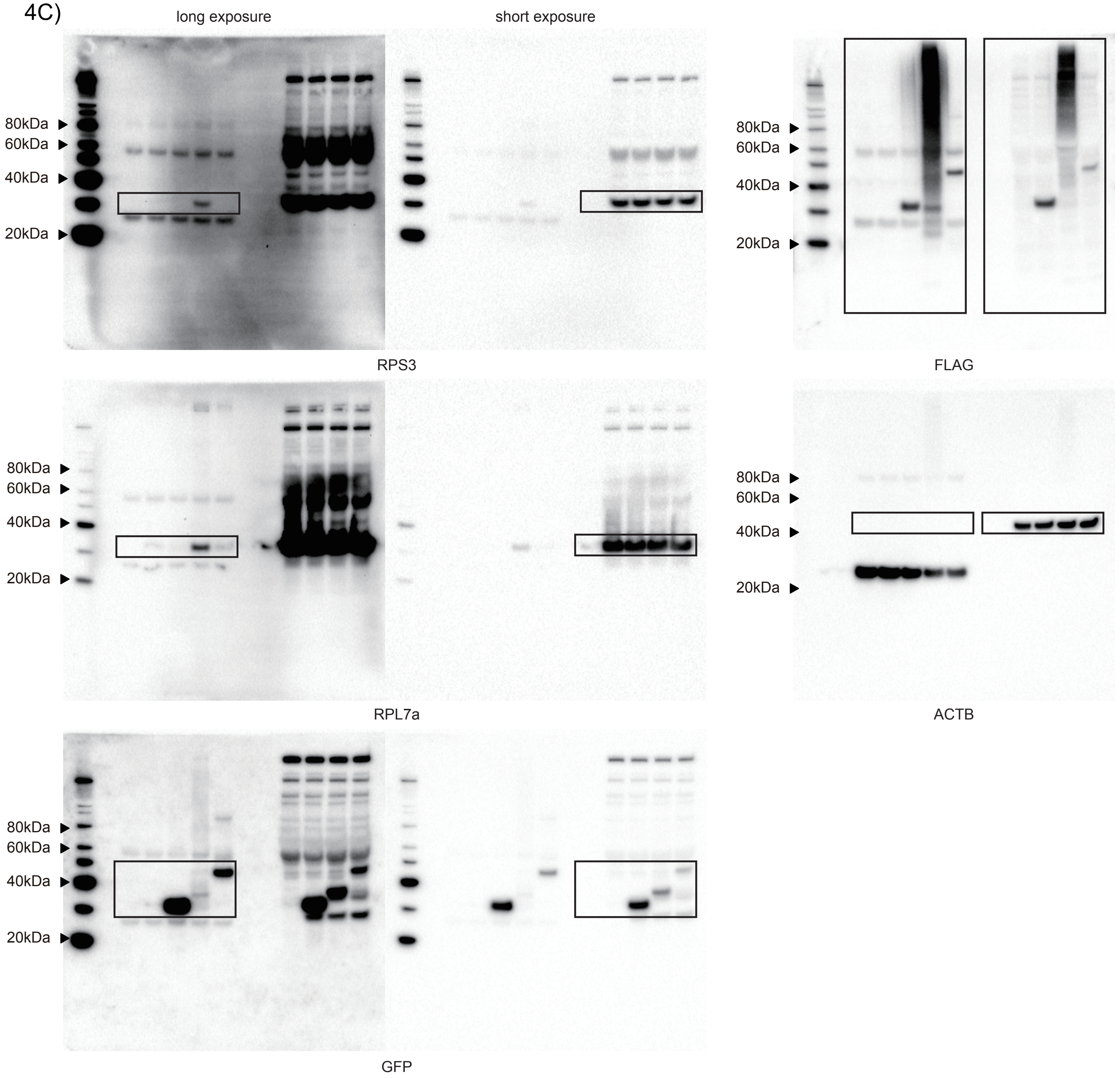
long exposure
short exposure
80kDa
60kDa
40kDa
20kDa
RPS3
80kDa
60kDa
40kDa
20kDa
FLAG
80kDa
60kDa
40kDa
20kDa
RPL7a
80kDa
60kDa
40kDa
20kDa
ACTB
80kDa
60kDa
40kDa
20kDa
GFP

4D)

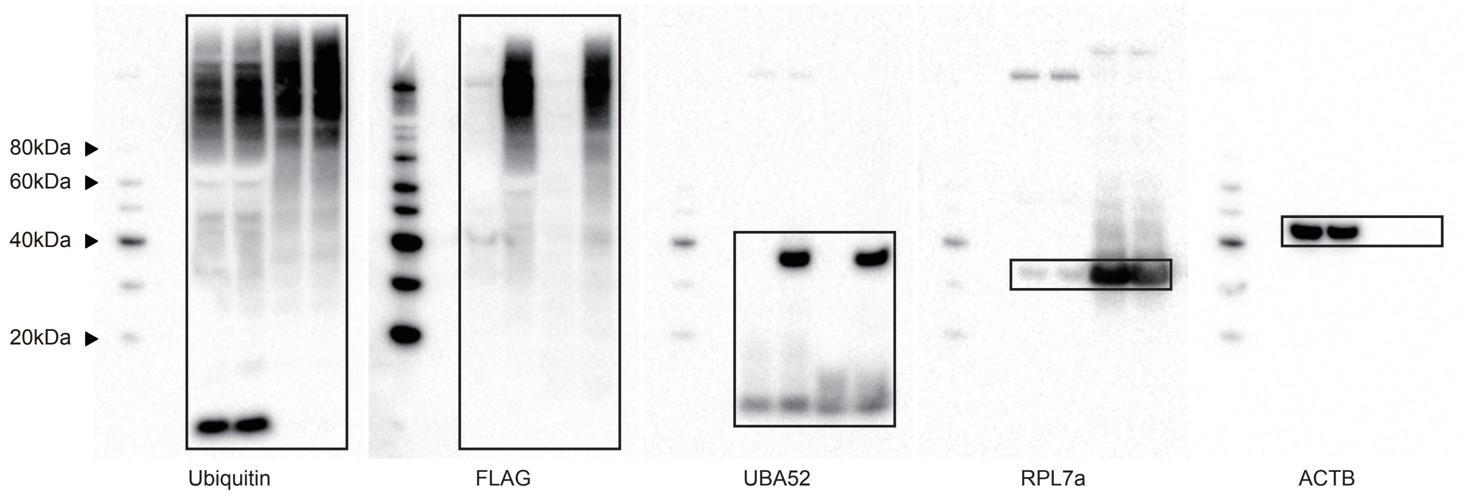
80kDa
60kDa
40kDa
20kDa
Ubiquitin
FLAG
UBA52
RPL7a
ACTB

4E)

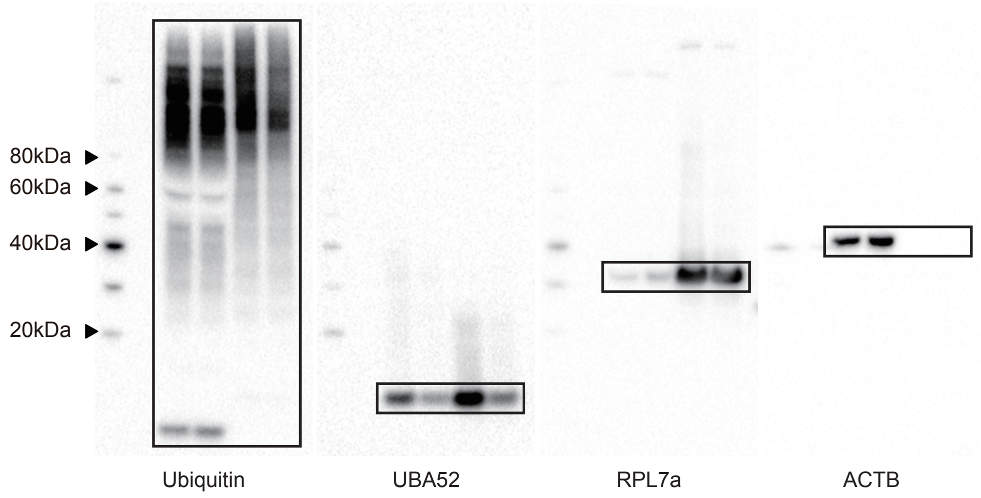
80kDa
60kDa
40kDa
20kDa
Ubiquitin
UBA52
RPL7a
ACTB

S1)

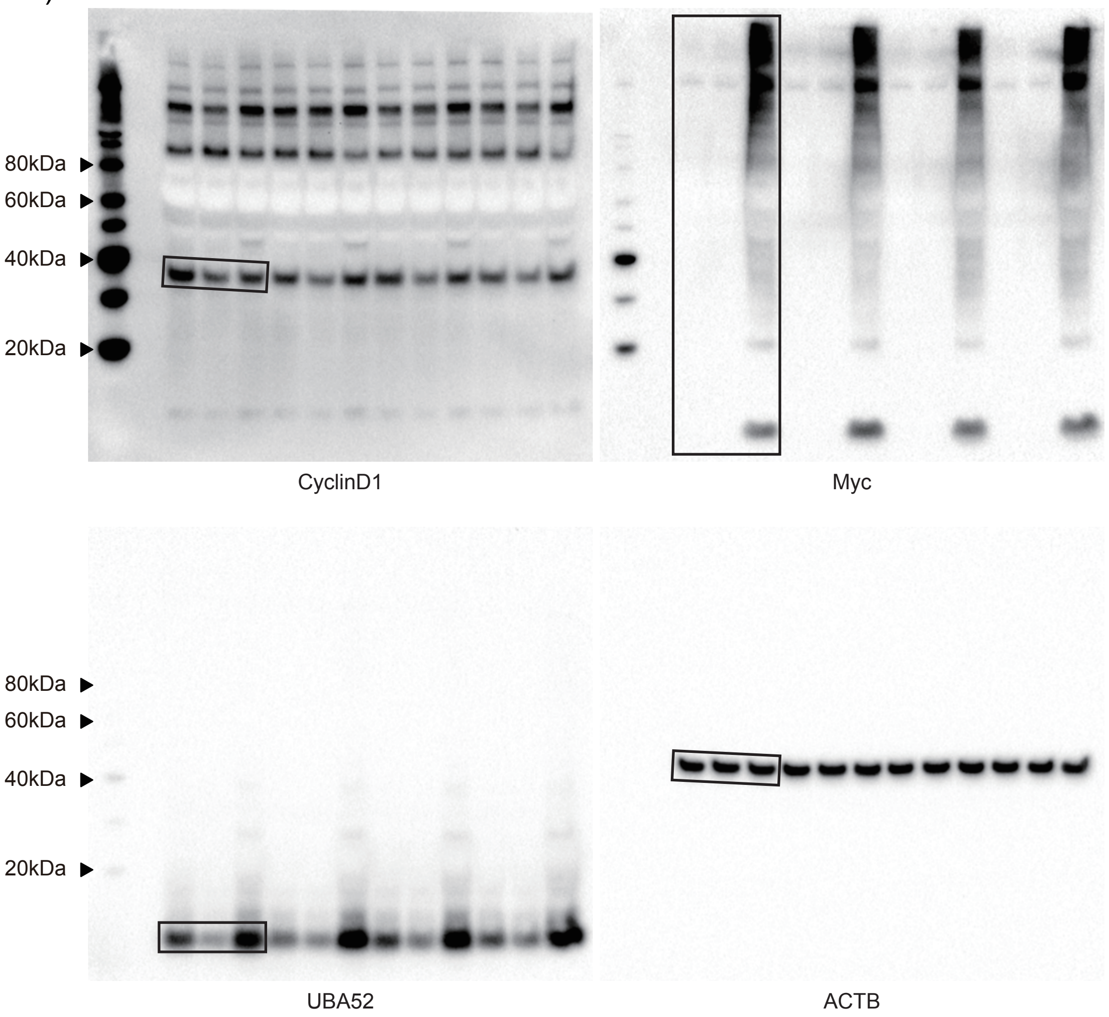
80kDa
60kDa
40kDa
20kDa
CyclinD1
Myc
80kDa
60kDa
40kDa
20kDa
UBA52
ACTB
